# MRI diffusion and perfusion alterations in the mesencephalon and pons as markers of disease and symptom reversibility in idiopathic normal pressure hydrocephalus

**DOI:** 10.1371/journal.pone.0240327

**Published:** 2020-10-08

**Authors:** Simon Agerskov, Jonathan Arvidsson, Doerthe Ziegelitz, Kerstin Lagerstrand, Göran Starck, Isabella M. Björkman-Burtscher, Carsten Wikkelsö, Mats Tullberg

**Affiliations:** 1 Department of Clinical Neuroscience, Hydrocephalus Research Unit, Institute of Neuroscience and Physiology, Sahlgrenska Academy, University of Gothenburg, Gothenburg, Sweden; 2 Department of Radiation Physics, Institute of Clinical Sciences, Sahlgrenska Academy, University of Gothenburg, Gothenburg, Sweden; 3 Department of Radiology, Institute of Clinical Sciences, Sahlgrenska Academy, University of Gothenburg, Gothenburg, Sweden; Goethe University Hospital Frankfurt, GERMANY

## Abstract

**Introduction:**

Core symptomatology in idiopathic normal pressure hydrocephalus (iNPH) points at dysfunction in the mesencephalon and pons indicating pathological changes in these regions, but only a few studies have addressed the issue. The aim of this study was to investigate diffusion (ADC) and perfusion patterns pre- and postoperatively in these areas in iNPH.

**Methods:**

Twenty iNPH patients and 15 healthy controls were included. Patients underwent a clinical examination and brain MRI pre- and 3–6 months postoperatively. The MRI-scan included diffusion and dynamic susceptibility contrast perfusion weighted sequences. Regions of interest in the mesencephalon and pons were drawn on a FLAIR sequence and co-registered to ADC maps and perfusion data.

**Results:**

There were no significant differences in pre or postoperative ADC compared to the control group, however postoperative ADC increased by 10% (p = 0.026) in the mesencephalon and 6% (p = 0.016) in the pons in all patients and also in the subgroup of shunt responders by 11% (p = 0.021) and 4% (p = 0.020), respectively. Preoperative relative cerebral blood flow (rCBF) was similar in iNPH patients and controls. Postoperatively, rCBF increased in shunt responders by 6% (p = 0.02) in the mesencephalon and 11% (p = 0.004) in the pons. This increase correlated with the degree of clinical improvement (r_s_ = 0.80, p = 0.031 and r_s_ = 0.66, p = 0.021, respectively).

**Conclusion:**

The postoperative increase in ADC and the correlation between postoperative increase in rCBF and clinical improvement in the mesencephalon and pons shown in this study point at an involvement of these areas in the core pathophysiology and its reversibility in iNPH.

## Introduction and aim

Idiopathic normal pressure hydrocephalus (iNPH) is a treatable neurological disease affecting the elderly [1, 2]. The clinical presentation is complex and may mimic effects of normal ageing, making the diagnosis difficult thereby contributing to the fact that iNPH is an under diagnosed and undertreated disease [3, 4].

MRI is essential in diagnosing iNPH, and a number of MRI-based biomarkers, both morphologic and functional, have been proposed as possible diagnostic and prognostic markers, albeit with conflicting results [1, 5–7].

Previous studies using diffusion and perfusion imaging have focused mainly on changes in supratentorial white matter regions reporting an increased preoperative ADC and mean diffusivity (MD) in iNPH with trends towards postoperative normalization [8–10]. A postoperative ADC increase in the basal ganglia has also been reported [11]. In addition, there is a correlation between the severity of clinical symptoms and a reduction in relative cerebral blood flow (rCBF) in periventricular white matter as well as central and frontal grey matter structures, together with a postoperative rCBF increase in shunt responders [12–15]. The rCBF increase in deep grey matter structures and in the gyrus cinguli has also been reported to correlate to the degree of postoperative clinical improvement [11, 13, 15].

Interestingly, core parts of the symptomatology in iNPH including disturbances in gait and postural control, cerebellar dystaxia, paratonia, impaired wakefulness and urinary urgency or incontinence, could represent dysfunction in systems neuroanatomically located in the mesencephalon and pons including the pedunculopontine nucleus in the mesencephalic tegmentum and the pontine micturition center [16–18]. So far, only a few radiological studies focusing on these regions have been performed [4, 9, 15, 19–25]. Results are inconsistent regarding morphological changes [21–23], and also from the few studies using diffusion and perfusion imaging techniques [9, 24]. Contrary to the ADC increase seen supratentorially [8–10], Tullberg et al. found no difference in ADC in periaqueductal grey matter between patients with iNPH and controls preoperatively [24]. Investigating changes in the corticospinal tract, Reiss-Zimmermann et al. found an increased MD in the fibres passing through the cerebral peduncles and the mesencephalon [9]. Relative cerebral blood flow has been reported to be reduced in the mesencephalon and cerebellum preoperatively using SPECT and PET-MRI [15, 25], in line with the reported changes in supratentorial structures, but no studies using MRI-perfusion techniques have been performed in these areas.

The aim of the present study was to characterize diffusion and perfusion patterns in iNPH in the mesencephalon and pons to analyse the importance of these structures for the symptomatology of the disease and their use as possible markers of response to shunt therapy.

## Materials and methods

### Study population

Twenty patients diagnosed with iNPH in accordance with international diagnostic guidelines [1], between 2005 and 2008, who underwent shunt surgery were compared to 15 healthy, age-matched controls recruited randomly from the population registry and the retired people’s organization, and examined during the same time interval and time of day. All patients and controls underwent complete imaging examinations as outlined below and none had structural defects in the mesencephalon and/or pons that could potentially influence the results. The population has previously been described in detail [12, 13], and demographic data are shown in Table 1.

**Table 1:** Demographic data for controls and iNPH patients (shunt responders and non-responders).

Demographic Data	Controls	iNPH patients		
	all	all	shunt responders	
	(n = 15)	(n = 20)	yes (n = 15)	no (n = 5)
Age in years, *mean (range)*	71 (64–82)	71 (55–82)	71 (59–82)	69 (55–80)
Sex, *n male/female (% males)*	9/6 (60)	12/8 (60)	8/7 (53)	4/1 (80)
Hypertension, *n yes/no (% yes)*	1/14 (7)	7/13 (35)	5/10 (33)	2/3 (40)
Duration of symptoms in months, *mean (range)*	n.a.	33 (7–168)	36 (7–168)	22 (18–24)
Preoperative Wahlund score for total WM changes, *mean (±SD)*	5 (±4.1)	6.2 (±4.8)	5.9 (±4.6)	7.2 (±7.8)
Preoperative Wahlund score for infratentorial WM changes, *mean (±SD)*	0.25 (±0.3)	0.33 (±0.4)	0.3 (±0.4)	0.4 (±0.5)
Preoperative Evans’ Index, *mean (±SD)*	n.a.	0.41 (±0.05)	0.40 (±0.06)	0.42 (±0.04)
iNPH Scale preoperative score, *mean (±SD)*	n.a.	52 (±24)	48 (±24)	65 (±23)
iNPH Scale postoperative score, *mean (±SD)*	n.a.	66 (±20)	68 (±19)	62 (±23)

iNPH = idiopathic Normal Pressure Hydrocephalus, WM = white matter, n.a. not applicable.

### Clinical testing and shunt surgery

All patients underwent detailed clinical testing pre- and 3–6 months postoperatively using a previously reported protocol [4]. Clinical symptoms were scored on a normalized outcome-scale, the iNPH scale by Hellström et al. [26], measuring gait, balance, cognitive and urinary function. A postoperative increase by ≥ 5 points was used as a cut off value for clinical improvement [26]. Clinical data are shown in Table 1.

All patients were operated with a ventriculo-peritoneal shunt, either the Codman Hakim^™^ Valve (Codman Johnson & Johnson, New Jersey 08933, USA) or the Strata^™^ Valve (Medtronic PS Medical, Santa Barbara, USA). Shunts were placed in the right frontal region except in one patient where it was placed in the right parietooccipital region.

### Imaging protocol

**Fig 1:**
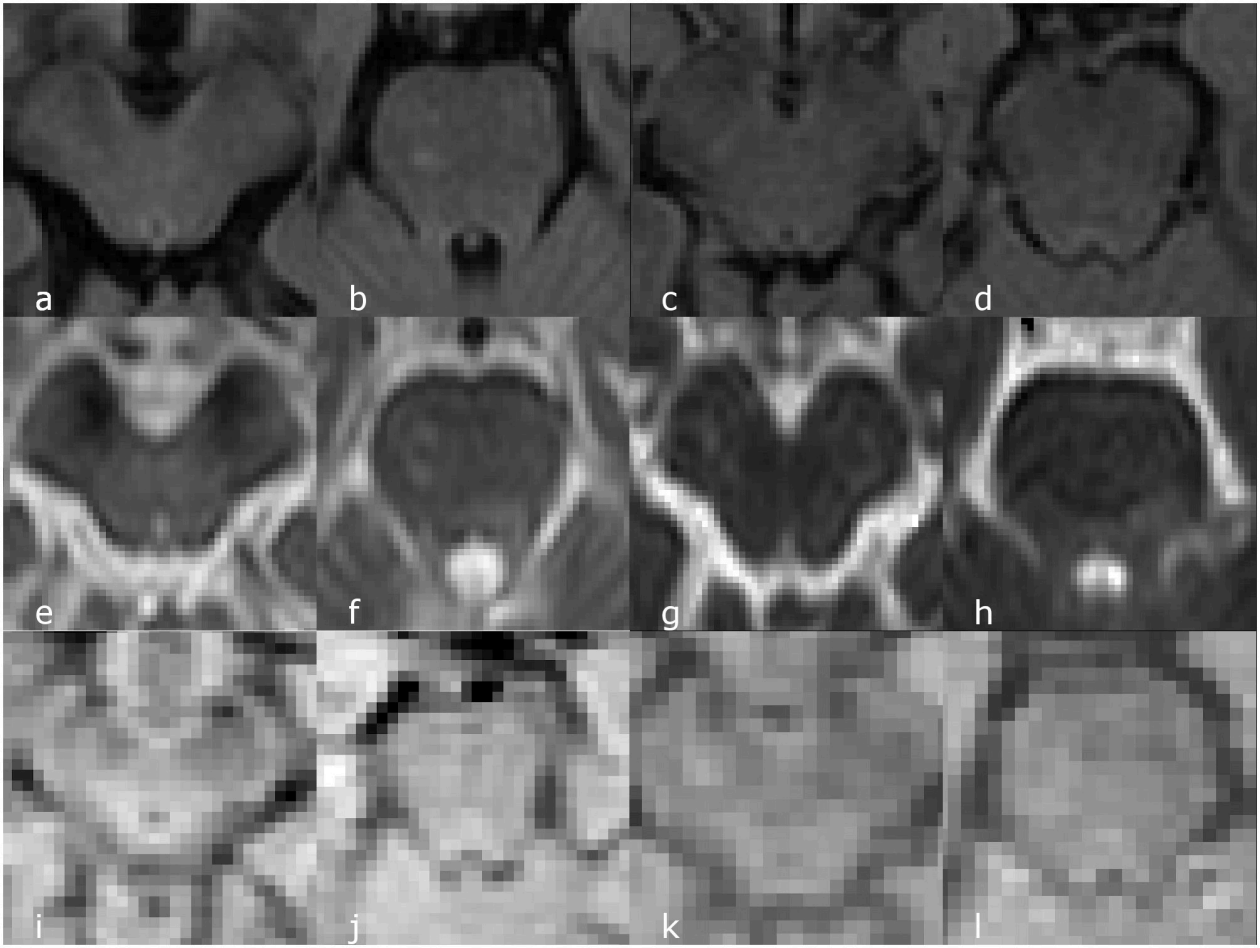
Example images. Example FLAIR images from a iNPH patient (a, b) and control (c, d) with corresponding ADC (e-h) and time averaged perfusion raw data (i-l) at the level of the mesencephalon and pons.

Pre- and postoperative MRI was performed on a 1.5T Gyroscan Intera 9.1 system (Philips Healthcare, Best, the Netherlands). The scan protocol has in part previously been reported and included morphological and quantitative perfusion sequences [12], as well as a quantitative diffusion weighted sequence. All sequences were angulated parallel to the callosal plane. The technical parameters are outlined below, and example images presented in Fig 1.

1. Transverse FLAIR sequence: TE 100 ms, TR 9000 ms, TI delay 2500 ms, slice thickness 3 mm, no slice gap, in-plane resolution 1.2 mm × 1.2 mm, FOV 230 mm× 230 mm, 192×192 acquisition matrix reconstructed to 256×256, pixel size 0.9 mm x 0.9 mm.
2. DSC perfusion: Images obtained every 1000 ms using a segmented k-space EPI technique: TE 30 ms, TR 500 ms, flip angle 40 degrees, 12 slices, slice thickness 5 mm, no slice gap, covering the posterior fossa and upper brainstem area, in-plane resolution 1.8×1.8 mm, FOV 230 mm× 230 mm, 128×128 matrix. At the tenth acquisition a 5 ml/s bolus of 0.1 mmol/kg Gd-DTPA (0.5 mmol/ml, Magnevist, Schering, Berlin, Germany) immediately followed by a 10 ml saline flush was administered in the right antecubital vein. The sequence was optimized for a high bandwidth in the phase encoding direction and scanning at a field strength of 1.5T rather than 3T to limit the postoperative, shunt-induced susceptibility effects.
3. Transverse DWI: TR 3793 ms, TE 90 ms, 25 slices, slice thickness 5 mm, no slice gap, in-plane resolution 1.2×2 mm, FOV 230 mm, acquisition matrix 192×113, b = 0 s/mm^2^ (1 acquisition) and b = 1000 s/mm^2^ (average of 3 signal acquisitions) in 3 orthogonal encoding directions. ADC maps with a 256×256 matrix and a pixel size of 0.9×0.9 mm were calculated. To reduce the sensitivity to motion, single shot EPI read out was used. No further motion correction was performed.

### Regions of interest

All ROIs for the perfusion- and diffusion evaluation were produced manually using ITK-Snap [27], by a resident in radiology (SA), and approved by a senior neuroradiologist (IBB) with more than 20 years of experience.

**Fig 2:**
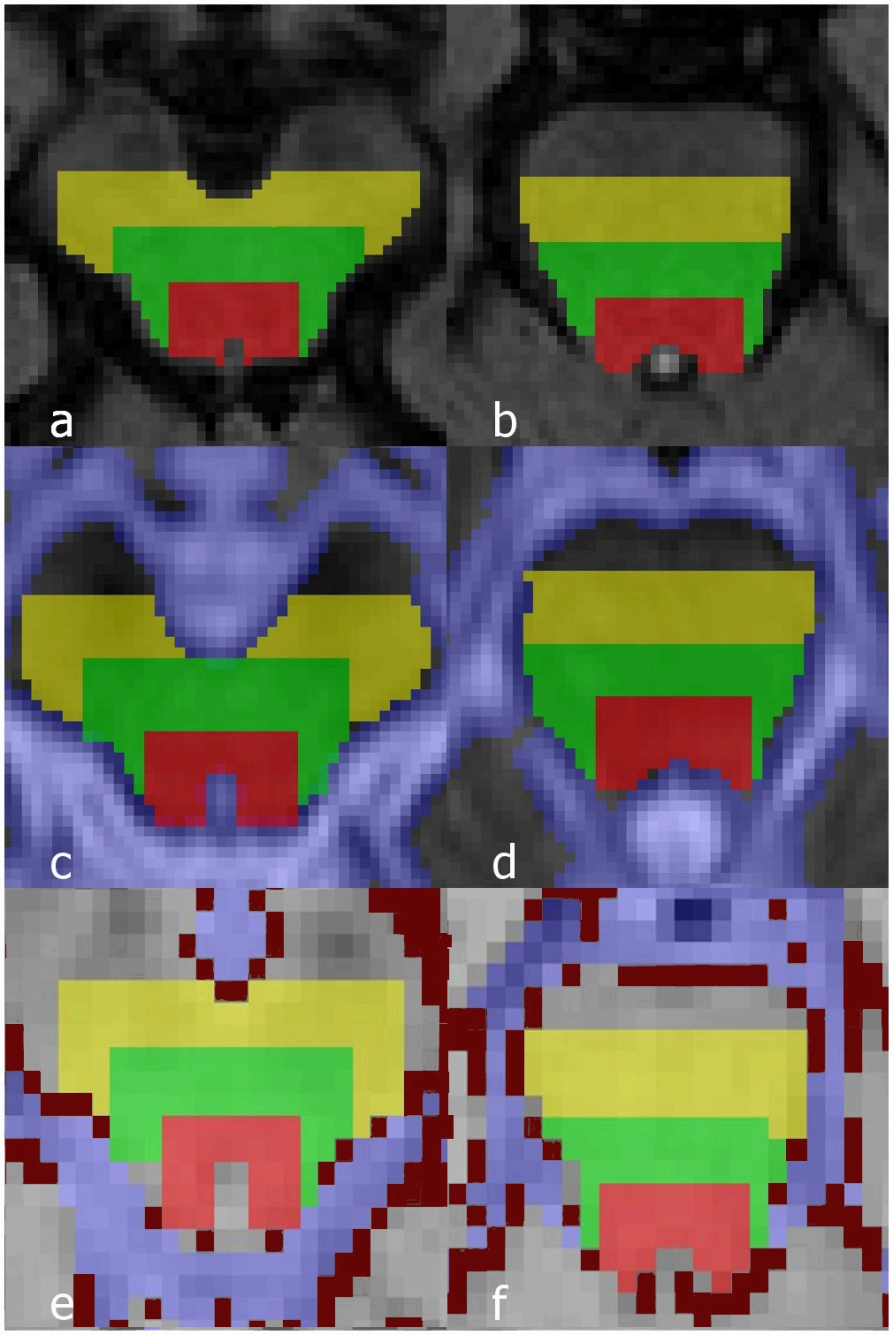
Example images after application of ROIs. FLAIR images at the level of the mesencephalon (a) and pons (b) after application of ROIs (red = posterior ROI, green = middle ROI, yellow = anterior ROI). Corresponding ADC (c, d) and time averaged perfusion raw data (e, f) after alignment and masking of the ROIs. Pale blue voxels (c-f) represent the CSF mask in both the ADC map and perfusion data and dark red voxels (e, f) represent the vessel mask in the perfusion data. Due to corrections for spatial differences and masking, the final ROI in the ADC and perfusion data differ slightly in shape compared to the original ROIs drawn on FLAIR images. Only one of two slices for each location is shown.

Regions of interest (ROIs) were drawn in two locations on FLAIR images, one in the upper part of the mesencephalon and one located approximately 6 mm (two slices) caudally in the pons (Fig 2). In each location three ROIs were drawn: a posterior, a middle and an anterior ROI, the middle and anterior ROIs radially expanding from the posterior ROI. ROIs were drawn on two consecutive slices in order to cover at least one slice in the lower resolution functional datasets. The posterior ROI at the level of the mesencephalon was centred around the anterior and lateral aspect of the cerebral aqueduct and at the pontine level aligned with the interface between the pons and the fourth ventricle. Each ROI was drawn to include a maximum anteroposterior depth of 6 pixels (approximately 5 mm), restricted by anatomical limitations in each in-plane direction with no overlap to the previous ROI. In addition, composite ROIs consisting of the three ROIs in the mesencephalon and pons respectively were created. ROI analysis was performed both for the composite ROIs and for the three ROIs in each location separately.

The FLAIR images were co-registered to the functional images and the ROIs were subsequently transferred. In the transfer, CSF areas in the FLAIR image were masked to ensure that the ROI was limited to brain parenchyma. In addition, a mask obtained using an in-house developed clustering approach was applied over the perfusion data to exclude large vessels [28]. Finally, to ensure that the ROIs drawn on the anatomical images were transferred correctly to the functional datasets, all transferred ROIs were manually checked and if necessary, small manual adjustments were made. The number of voxels in each final ROI adapted to anatomical structures was recorded (S1 Table). Fig 2 displays examples of the final ROIs after co-registration to the functional datasets. For internal reference, ROIs delineating grey matter in the occipital cortex were used [12].

The ROI processing and co-registration was performed using an in-house developed batch-processing pipeline written in MATLAB 2018b (The MathWorks, Natick, 2018), utilizing parts of the software packages SPM12 (Functional Imaging Laboratory, The Wellcome Trust Centre for NeuroImaging, UK, 2014) and NiftyReg (School of Biomedical Engineering and Imaging Sciences, King’s College London, The United Kingdom) [29].

### Extraction of diffusion estimates–ADC

ADC maps, displaying ADC estimates pixel- by-pixel, were reconstructed using the standard scanner software or an in-house developed software. To calculate the ADC estimates, the b0 and b1000 images of the DWI examination were used, where a mono-exponential diffusion model (SDWI = S0e (-b ADC)) including the two b-values enabled a closed form expression for the ADC estimates [30]. A validation set consisting of five patients that had both DWI and ADC data archived was used to evaluate the consistency between the two processing platforms (S1 Fig). Finally, the reconstructed ADC maps were given as input to the in-house batch-processing pipeline, producing a mean ADC value for each ROI.

### Extraction of perfusion estimates–rCBF, rCBV and MTT

The arterial input function (AIF) was manually selected using an in-house developed software. The selection criteria for the AIF-voxels were, as previously described [12, 30], an early time of bolus arrival, a steep initial signal decrease, and a deep signal dip. Moreover, 1–4 voxels that fulfilled these criteria were selected and averaged to improve the signal to noise ratio (SNR) of the AIF. De-convolution of the signal-time-curves with the AIF was performed using an in-house developed software, implementing the vascular model-based single compartment de-convolution technique [31]. The model was fitted, in accordance with the original publication, via a Bayesian cost function and a Maximum Likelihood Expectation Maximization optimization scheme. Deconvolution was performed on the ROI average signal-time-curves. Relative cerebral blood flow (rCBF) and relative cerebral blood volume (rCBV) measures were extracted by normalizing each CBF- or CBV-estimate with that of grey matter using the reference ROIs in the occipital lobe [12]. Relative perfusion measures were used instead of absolute values in order to reduce the random and nonlinear scaling of CBF and CBV estimates caused by partial volume effects of the AIF-voxels [32]. Mean transit time (MTT) was obtained using the central volume theory as the ratio of CBV/CBF [33]. The in-house developed batch-processing pipeline was used for the perfusion analysis.

### Statistical analysis

Nonparametric statistical tests were used. Differences in distributions among binary variables were tested using Pearson chi^2^ or Fishers’ exact test. Tests of differences between groups for ordinal and interval data were performed using the Wilcoxon rank sum test. Distributions over time in binary variables were tested using the paired samples McNemar test while ordinal and continuous variables were tested using the Wilcoxon signed rank test Correlations were tested using Spearman rank correlations. Significance was set at p < .05 (two-tailed). Due to the low number of non-responders, no statistical tests were performed for this group. No corrections for multiple comparisons were made. All calculations were performed using the software SPSS, Version 24.0 (IBM Corp. Released 2016. IBM SPSS Statistics for Windows, Version 24.0. Armonk, NY).

### Ethical considerations

The study was approved by the regional ethical review board at Gothenburg University (DNR: 154–05) and written informed consent was obtained from all participants or close relatives.

## Results

Seventy-five % (15/20) of patients improved ≥ 5 points on the iNPH scale after surgery (responders). One patient in the non-responder group had developed a small, right sided, chronic subdural hematoma at the postoperative control which was considered asymptomatic and the patient was included in the analyses. Four patients’ postoperative scans were excluded due to visible susceptibility artefacts.

### Diffusion

There were no differences in pre- or postoperative ADC values in iNPH patients compared to controls (Table 2). However, a postoperative ADC increase was seen in the mesencephalon and the pons in the whole group and in responders. Pre- or postoperative ADC values did not correlate with the pre or postoperative iNPH scale score in the whole sample or in either subgroup, nor did the postoperative change in ADC values correlate with the postoperative change in iNPH scale score (data not shown).

**Table 2:** Apparent diffusion coefficient (ADC) values in controls and iNPH patients given for the composite ROI in the mesencephalon and pons.

Group		ADC 10^-6^mm^2^/s, *median (IQR) % of control value*			
		Brain region			
		Mesencephalon	P	Pons	P
Controls (n = 15)		795 (765–836) n.a.		769 (746–872) n.a.	
All iNPH patients	Preoperative (n = 20)	791 (752–820) 99	0.48^a^	771 (736–802) 100	0.24^a^
	Postoperative (= 16)	851 (789–875) 107	0.26^a^	781 (756–859) 102	0.31^a^
	Change (n = 16)	80 (-45-109) 10	0.026^b^	43 (-13-71) 6	0.016^b^
Responders	Preoperative (n = 15)	800 (750–821) 100	0.62^a^	772 (738–813) 100	0.44^a^
	Postoperative (n = 12)	856 (844–878) 108	0.51^a^	779 (757–888) 102	0.32^a^
	Change (n = 12)	88 (15–118) 11	0.021^b^	32 (-13-71) 4	0.020^b^
Non-responders	Preoperative (n = 5)	775 (753–828) 97		760 (721–798) 98	
	Postoperative (n = 4)	797 (750–842) 100		773 (755–803) 100	
	Change (n = 4)	-39 (-69-88) -5		63 (-50-72) 8	

^a^compared to control values,

^b^compared to preoperative values,

IQR = Interquartile Range.

In the analysis of the smaller ROIs (posterior, middle, anterior), similarly to the composite ROIs, no significant differences in pre- or postoperative ADC-values compared to controls were seen. A small, but significant postoperative ADC increase was seen in the middle mesencephalic as well as middle and anterior pontine ROIs (S2 Table).

### Perfusion

**Fig 3:**
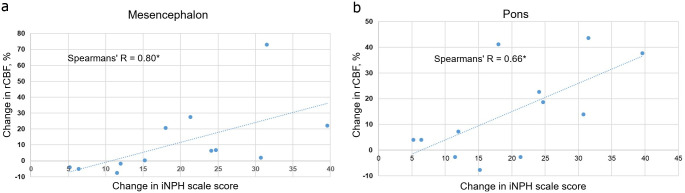
Correlations between rCBF and iNPH scale score. Correlations between change in relative cerebral blood flow (rCBF) in the mesencephalon (a) and pons (b) and change in the iNPH scale score after surgery in responders.

There were no significant differences in rCBF between iNPH-patients and controls prior to surgery. Postoperatively, rCBF increased in both the mesencephalon and pons in the responder group whereas in non-responders, a numeric rCBF decrease was seen in the mesencephalon (Table 3). Pre- or postoperative rCBF values did not correlate with the pre or postoperative iNPH scale score in the whole sample or in either subgroup, however, the increase in rCBF after surgery in both the mesencephalon and pons correlated to the degree of change of the iNPH scale score in responders, r_s_ = 0.80 (p = 0.031) and r_s_ = 0.66 (p = 0.021) respectively (Fig 3a and 3b).

**Table 3:** Relative cerebral blood flow (rCBF) values in controls and iNPH patients given for the composite ROI in the mesencephalon and pons.

Group		rCBF, *median (IQR) % of control value*			
		Brain region			
		Mesencephalon	P	Pons	P
Controls (n = 15)		0.84 (0.79–0.91)		0.72 (0.67–0.81)	
All iNPH patients	Pre-OP (n = 20)	0.86 (0.77–0.97) 101	0.91^a^	0.69 (0.64–0.81) 95	0.48^a^
	Post-OP (= 16)	0.91 (0.81–0.98) 106	0.38^a^	0.82 (0.71–0.86) 111	0.11^a^
	Change (n = 16)	0.02 (-0.1–0.11) 2	0.64^b^	0.06 (-0.02–0.16) 7	0.07^b^
Responders	Pre-OP (n = 15)	0.86 (0.77–0.93) 101	0.78^a^	0.69 (0.61–0.77) 95	0.27^a^
	Post-OP (n = 12)	0.93 (0.88–1.01)109*	0.039^a^	0.83 (0.73–0.86)113	0.023^a^
	Change (n = 12)	0.05 (0.01–0.16) 6	0.034^b^	0.07 (0.04–0.21) 11^a^^,^^b^	0.002^b^
Non-responders	Pre-OP (n = 5)	0.90 (0.74–1.02) 106		0.77 (0.68–0.94) 104	
	Post-OP (n = 4)	0.78 (0.67–0.87) 92		0.73 (0.61–0.85) 100	
	Change (n = 4)	-0.12 (-0.17–-0.02) -14		-0.05 (-0.2–-0.02) -7	

^a^compared to control values,

^b^compared to preoperative values, = Interquartile Range. OP, operative.

There were no significant differences in rCBF in the posterior, middle or anterior ROIs between iNPH patients and controls. Postoperatively, rCBF increased significantly in the middle mesencephalic and pontine ROIs in the responder group (S3 Table). The rate of rCBF increase in the middle pontine ROI correlated significantly to the degree of clinical improvement in responders (r_s_ = 0.69, p = 0.013).

There were no significant differences in rCBV or MTT in the iNPH patient group compared to controls nor any significant postoperative changes or correlation to clinical improvement.

## Discussion

In this study, both ADC and rCBF increased in responders postoperatively in the pons and mesencephalon with the rate of rCBF increase correlating to the degree of clinical improvement. Even though no differences in ADC or rCBF were found between iNPH-patients and controls at baseline, our postoperative findings indicate that functional changes in the mesencephalon and pons are involved in disease development and symptom generation of iNPH. The findings also further support the view that estimates of rCBF using DSC-MRI reflect clinical reversibility.

Similarly to a previous study by Tullberg et al. [24], but differing from several studies investigating supratentorial white matter areas in iNPH [8, 11, 34, 35], the present study could not demonstrate significant differences in ADC between iNPH-patients and controls pre- or postoperatively. Disease mechanisms resulting in an ADC-increase such as an increased water content in the extracellular space might be more strongly expressed in the white matter of iNPH patients than in grey matter areas, both supratentorially and in the midbrain and thus, the inclusion of both grey and white matter in the ROIs used in the present study might explain our results [9, 36]. Similarly to ADC findings in the basal ganglia reported by Tuniz et al., we found a significant postoperative ADC increase in the patient group [11]. Even though the preoperative ADC-levels reported did not differ from the control group values, the postoperative increase could be caused by a relief of chronic ischemia, potentially due to decompression similar to what has been previously reported in the corpus callosum [37], affecting grey matter in the brainstem and pons [11].

In previous studies, also including data from this cohort, iNPH-patients showed a decrease in rCBF compared to control subjects, most pronounced in the medial frontal cortex and in periventricular structures [12–15], suggesting an altered metabolism in periventricular areas [12], and/or microvascular dysfunction in iNPH [14]. This study, using rCBF measurements, did not show significant differences, possibly due to a global perfusion impairment in iNPH affecting the internal reference ROI as well [12, 15], causing relative perfusion values to be overestimated and masking a between-group perfusion difference.

Postoperatively, rCBF increased significantly in responders with strong correlations to the magnitude of clinical improvement (Fig 3a and 3b), while a further reduction was seen in the non-responder group (Table 3). Similar results have been reported previously in both the mesencephalon [15], and supratentorial structures [11, 13], with one previous study using the same dataset as the present study [13]. The postoperative rCBF increase in the mesencephalon and pons to a higher level than in controls reported here supports the claim that a postoperative restoration, probably due to an increased metabolism in these areas is important for re-establishing normal physiological functions after surgery [13]. A larger postoperative rCBF increase in these areas compared to the reference ROIs could explain why levels are higher than in controls. The postoperative rCBF reduction in the mesencephalon in non-responders could hypothetically be explained by a preoperative CBF reduction in the reference ROIs with postoperative restoration of CBF in this area, but not in the mesencephalon, resulting in a decrease of the mesencephalic rCBF [38].

The postoperative increase in rCBF in responders was seen only in the middle ROI in both the mesencephalon and pons, correlating to clinical improvement (S3 Table). These findings imply that metabolic and/or vascular changes in iNPH are most prominent in regions outside of the absolute periventricular region which is supported by a previous study [14], showing CBF reduction after a CSF-infusion test, where the reduction was most prominent approximately 9 mm from the ventricular wall.

### Strengths and limitations

The main strengths of this study are the clinically well characterized patient group and the use of a single MRI scanner for both the pre- and postoperative examination of the patients as well as the control population. Further, the design of the DSC-MRI pulse sequence and the choice of a 1.5T machine minimized shunt artefacts making it possible to obtain perfusion estimates postoperatively.

This study also has limitations. The small number of patients and controls introduces a risk that differences between groups or postoperative changes are not detected. Repeated MR examinations might also have been advantageous for control subjects in order to rule out other factors than postsurgical response causing the observed postoperative rCBF and ADC changes. However, to avoid an additional contrast administration and ethical issues related to such a procedure, the study design did not allow for this approach. The fact that patients and controls were examined using the same MRI scanner with identical protocols (for the patient group this was done pre- and postoperatively), during the same time period and at the same time of day should still limit this risk. A third limitation are the few diffusion vectors used in this study that result in a lower sensitivity for ADC changes. Fourth, although patients who had visual shunt artefacts in the postoperative scans in the areas of interest were excluded, we cannot rule out influence of shunt artefacts on the postoperative results. However, postoperative changes in ADC and the anatomical distance between the shunt and areas of interest in this study are in line with previously published results [10, 24]. The use of two different methods for calculating ADC is another weakness, but the high consistency between these methods should limit the potential bias.

Further, DSC MRI is prone to high noise levels in extracted concentration-time curves. Consequently, despite the use of a sophisticated deconvolution technique, derived perfusion parameter estimates will in relation to true values be associated with a bias and a variability. Moreover, the calculation of relative CBF values depends on signal to concentration transform which can be a potential source of error during data analysis. These errors may limit the ability to diagnose individual patients with iNPH. On a group level, however, the method has provided estimates that correlated with postoperative symptom relief. With a larger population, DSC MRI may have the potential to help separate subgroups within iNPH e.g. responders and non-responders.

Finally, the prevalence of hypertension was higher in the patient group compared to controls which could potentially influence the results. However, the absence of significant structural changes in the mesencephalon and pons as well as the similar prevalence of white matter lesions in infratentorial structures in both groups indicate that the potential effect in these areas is small.

## Conclusions

The postoperative increase in ADC and the correlation between postoperative increase in rCBF and clinical improvement in the mesencephalon and pons point at involvement of these areas, in addition to supratentorial structures in the core pathophysiology and its reversibility in iNPH. These areas should be additional targets in further studies of the pathophysiology of the disease.

## Supporting information

S1 Fig Plot showing the correlation between the two methods for ADC calculation. (TIF)

S1 Table Range of voxels in the final ROIs after alignment and masking. (DOCX)

S2 Table Apparent diffusion coefficient (ADC) values in controls and iNPH patients given for the individual posterior, middle and anterior ROIs in the mesencephalon and pons. (DOCX)

S3 Table Relative cerebral blood flow (rCBF) values in controls and iNPH patients given for the individual posterior, middle and anterior ROIs in the mesencephalon and pons. (DOCX)

S1 File Core data set. (SAV)
