# Supplementary figures and images for: MRI diffusion and perfusion alterations in the mesencephalon and pons as markers of disease and symptom reversibility in idiopathic normal pressure hydrocephalus
Source: PLoS One. 2020 Oct 8;15(10):e0240327. doi: 10.1371/journal.pone.0240327 (PMC7544092; doi:10.1371/journal.pone.0240327)

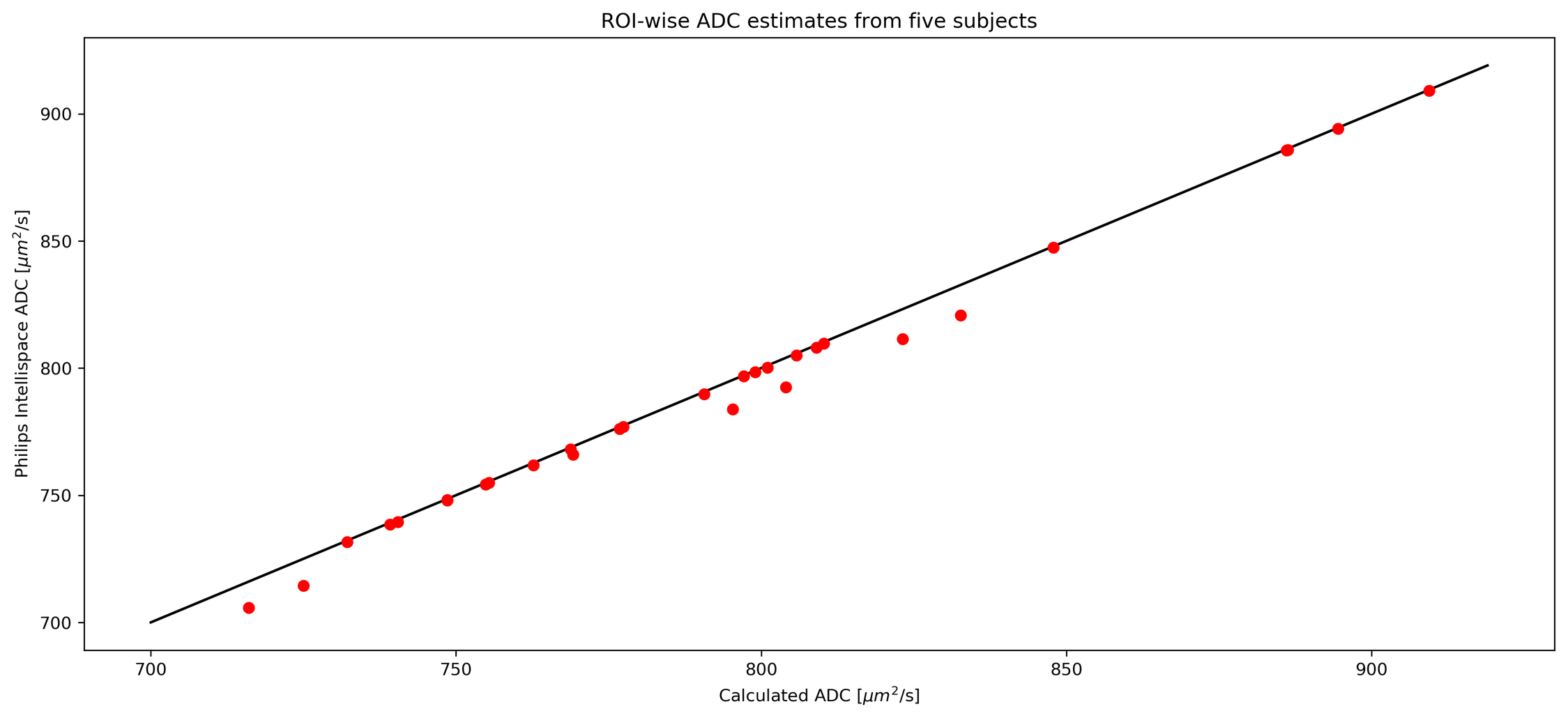

Supplement: S1 Fig — (TIF) [file pone.0240327.s001.tif]
