# Supplementary material for: MRI diffusion and perfusion alterations in the mesencephalon and pons as markers of disease and symptom reversibility in idiopathic normal pressure hydrocephalus
Source: PLoS One. 2020 Oct 8;15(10):e0240327. doi: 10.1371/journal.pone.0240327 (PMC7544092; doi:10.1371/journal.pone.0240327)
Supplement: S1 Table — (DOCX) [file pone.0240327.s002.docx]

|  |  | Range of voxels after alignment and masking | | |
| --- | --- | --- | --- | --- |
| **ADC** |  |  | Brain region | |
| Group |  | ROI | Mesencephalon | Pons |
| Controls (n=15) |  | Posterior | 142-343 | 124-387 |
|  |  | Middle | 254-560 | 212-539 |
|  |  | Anterior | 214-632 | 152-491 |
| All iNPH patients | Preoperative (n=20) | Posterior | 109-343 | 96-387 |
|  |  | Middle | 213-560 | 197-539 |
|  |  | Anterior | 239-632 | 160-491 |
|  | Postoperative (n=16) | Posterior | 88-426 | 111-407 |
|  |  | Middle | 199-544 | 231-599 |
|  |  | Anterior | 207-570 | 155-631 |
| **Perfusion data** |  |  | Brain region | |
| Group |  | ROI | Mesencephalon | Pons |
| Controls (n=15) |  | Posterior | 47-111 | 41-99 |
|  |  | Middle | 64-168 | 55-157 |
|  |  | Anterior | 83-205 | 65-143 |
| All iNPH patients | Preoperative (n=20) | Posterior | 54-111 | 40-89 |
|  |  | Middle | 66-168 | 56-145 |
|  |  | Anterior | 84-184 | 71-143 |
|  | Postoperative (n=16) | Posterior | 50-113 | 41-105 |
|  |  | Middle | 69-163 | 54-150 |
|  |  | Anterior | 95-185 | 54-146 |

**S1 Table.** Range of voxels in the final ROIs after alignment and masking
