# Supplementary material for: MRI diffusion and perfusion alterations in the mesencephalon and pons as markers of disease and symptom reversibility in idiopathic normal pressure hydrocephalus
Source: PLoS One. 2020 Oct 8;15(10):e0240327. doi: 10.1371/journal.pone.0240327 (PMC7544092; doi:10.1371/journal.pone.0240327)
Supplement: S2 Table — (DOCX) [file pone.0240327.s003.docx]

**S2 Table.** Apparent diffusion coefficient (ADC) values in controls and iNPH patients given for the individual posterior, middle and anterior ROIs in the mesencephalon and pons

|  |  |  | ADC 10^-6^mm^2^/s, *median (IQR) % of control value* | | | |
| --- | --- | --- | --- | --- | --- | --- |
| ROI | Group |  | Brain region | | | |
|  |  |  | Mesencephalon | P | Pons |  |
| Posterior |  |  |  |  |  |  |
|  | Controls (n=15) |  | 820 (781-877) n.a. |  | 787 (748-942) n.a. |  |
|  | All iNPH patients | Preoperative (n=20) | 798 (760-861) 97 | 0.42^a^ | 805 (764-836) 102 | 0.88^a^ |
|  |  | Postoperative (=16) | 867 (793-905) 105 | 0.39^a^ | 798 (774-824) 101 | 0.81^a^ |
|  |  | Change (n=16) | 53 (-37-148) 6 | 0.11^b^ | 4 (-14-63) -1 | 0.30^b^ |
|  | Responders | Preoperative (n=15) | 832 (744-865) 101 | 0.60^a^ | 806 (771-827) 102 | 0.90^a^ |
|  |  | Postoperative (n=12) | 892 (798-909) 108 | 0.09^a^ | 799 (775-853) 101 | 0.82^a^ |
|  |  | Change (n=12) | 89 (-34-179) 11 | 0.08^b^ | 1 (-13-74) 0 | 0.42^b^ |
|  | Non-responders | Preoperative (n=5) | 796 (763-832) 97 |  | 748 (743-865) 95 |  |
|  |  | Postoperative (n=4) | 841 (751-862) 102 |  | 797 (740-811) 101 |  |
|  |  | Change (n=4) | -18 (-48-65) -2 |  | 57 (-150-178) 7 |  |
| Middle |  |  |  |  |  |  |
|  | Controls (n=15) |  | 811 (759-833) n.a. |  | 786 (737-864) n.a. |  |
|  | All iNPH patients | Preoperative (n=20) | 780 (738-816) 96 | 0.30^a^ | 758 (716-791) 96 | 0.08^a^ |
|  |  | Postoperative (=16) | 815 (782-887) 100 | 0.21^a^ | 763 (751-808) 97 | 0.07^a^ |
|  |  | Change (n=16) | 65 (-26-85) 8 | 0.04^b^ | 34 (-5-81) 4 | 0.026^b^ |
|  | Responders | Preoperative (n=15) | 801 (741-817) 98 | 0.49^a^ | 754 (713-797) 95 | 0.09^a^ |
|  |  | Postoperative (n=12) | 823 (805-890) 101 | 0.38^a^ | 762 (750-864) 97 | 0.08^a^ |
|  |  | Change (n=12) | 73 (28-96) 9 | 0.021^b^ | 35 (-3-86) 4 | 0.021^b^ |
|  | Non-responders | Preoperative (n=5) | 751 (736-816) 92 |  | 769 (717-787) 98 |  |
|  |  | Postoperative (n=4) | 784 (753-871) 97 |  | 764 (755-823) 97 |  |
|  |  | Change  (n=4) | -17 (-77-102) -2 |  | 19 (-40-129) 2 |  |
| Anterior |  |  |  |  |  |  |
|  | Controls (n=15) |  | 768 (738-820) n.a. |  | 777 (752-855) n.a. |  |
|  | All iNPH patients | Preoperative (n=20) | 786 (747-808) 102 | 0.82^a^ | 749 (712-780) 96 | 0.075^a^ |
|  |  | Postoperative (=16) | 804 (762-876) 105 | 0.79^a^ | 771 (742-838) 99 | 0.07^a^ |
|  |  | Change (n=16) | 27 (-29-150) 4 | 0.12^b^ | 53 (3-112) 7 | 0.02^b^ |
|  | Responders | Preoperative (n=15) | 780 (747-806) 101 | 0.74^a^ | 765 (716-816) 98 | 0.21^a^ |
|  |  | Postoperative (n=12) | 828 (769-886) 108 | 0.69^a^ | 773 (739-835) 99 | 0.24^a^ |
|  |  | Change (n=12) | 31 (-18-170) 4 | 0.08^b^ | 54 (-35-104) 7 | 0.07^b^ |
|  | Non-responders | Preoperative (n=5) | 800 (745-842) 104 |  | 729 (703-761) 94 |  |
|  |  | Postoperative (n=4) | 762 (748-832) 99 |  | 769 (747-799) 98 |  |
|  |  | Change  (n=4) | 57 (-150-135) 7 |  | 52 (40-132) 6 |  |

Note: ^a^compared to control values, ^b^compared to preoperative values, IQR = Interquartile Range.
