# Supplementary material for: MRI diffusion and perfusion alterations in the mesencephalon and pons as markers of disease and symptom reversibility in idiopathic normal pressure hydrocephalus
Source: PLoS One. 2020 Oct 8;15(10):e0240327. doi: 10.1371/journal.pone.0240327 (PMC7544092; doi:10.1371/journal.pone.0240327)
Supplement: S3 Table — (DOCX) [file pone.0240327.s004.docx]

**S3 Table.** Relative cerebral blood flow (rCBF) values in controls and iNPH patients given for the individual posterior, middle and anterior ROIs in the mesencephalon and pons

|  |  |  |  | rCBF, *median (IQR) % of control value* | |  |
| --- | --- | --- | --- | --- | --- | --- |
| ROI | Group |  |  | Brain region | |  |
|  |  |  | Mesencephalon | P | Pons | P |
| Posterior |  |  |  |  |  |  |
|  | Controls (n=15) |  | 0.74 (0.68-0.83) |  | 0.56 (0.53-0.63) |  |
|  | All iNPH patients | Preoperative (n=20) | 0.80 (0.71-0.91) 108 | 0.52^a^ | 0.55 (0.43-0.60) 98 | 0.31^a^ |
|  |  | Postoperative (n=16) | 0.83 (0.73-1.01) 112 | 0.14^a^ | 0.61 (0.52-0.79) 109 | 0.31^a^ |
|  |  | Change  (n=16) | 0.03 (-0.12-0.22) 4 | 0.38^b^ | 0.06 (-0.01-0.19) 11 | 0.09^b^ |
|  | Responders | Preoperative (n=15) | 0.81 (0.70-0.91) 109 | 0.56^a^ | 0.53 (0.43-0.57) 95 | 0.07^a^ |
|  |  | Postoperative (n=12) | 0.83 (0.74-1.01) 112 | 0.08^a^ | 0.61 (0.52-0.73) 109 | 0.51^a^ |
|  |  | Change  (n=12) | 0.02 (-0.10-0.25) 3 | 0.27^b^ | 0.08 (0.04-0.21) 14 | 0.09^b^ |
|  | Non-responders | Preoperative  (n=5) | 0.77 (0.67-0.91) 104 |  | 0.66 (0.57-0.72) 117 |  |
|  |  | Postoperative  (n=4) | 0.77 (0.61-0.95) 104 |  | 0.70 (0.50-0.88) 125 |  |
|  |  | Change  (n=4) | 0.00 (-0.15-0.17) 0 |  | 0.04 (-0.07-0.11) 8 |  |
| Middle |  |  |  |  |  |  |
|  | Controls (n=15) |  | 0.84 (0.77-0.91) |  | 0.78 (0.72-0.89) |  |
|  | All iNPH patients | Preoperative (n=20) | 0.83 (0.76-0.95) 99 | 0.78^a^ | 0.73 (0.67-0.88) 94 | 0.35^a^ |
|  |  | Postoperative (n=16) | 0.84 (0.73-0.94) 100 | 0.89^a^ | 0.85 (0.71-0.94) 109 | 0.37^a^ |
|  |  | Change (n=16) | 0.01 (-0.06-0.12) 1 | 0.67^b^ | 0.12 (0.05-0.21) 15 | 0.21^b^ |
|  | Responders | Preoperative (n=15) | 0.82 (0.75-0.88) 98 | 0.62^a^ | 0.70(0.63-0.86) 90 | 0.21^a^ |
|  |  | Postoperative (n=12) | 0.88 (0.81-0.98) 105 | 0.09^a^ | 0.87 (0.79-0.96) 112 | 0.08^a^ |
|  |  | Change (n=12) | 0.06 (0.01-0.17) 7 | 0.039^b^ | 0.17 (0.03-0.25) 22 | 0.015^b^ |
|  | Non-responders | Preoperative (n=5) | 0.85 (0.73-1.03) 102 |  | 0.83 (0.72-1.01) 106 |  |
|  |  | Postoperative (n=4) | 0.67 (0.59-0.76) 80 |  | 0.67 (0.62-0.77) 86 |  |
|  |  | Change  (n=4) | -0.17 (-0.29- -0.08) -22 |  | -0.16 (-0.26-0.02) -20 |  |
| Anterior |  |  |  |  |  |  |
|  | Controls (n=15) |  | 0.89 (0.81-1.1) |  | 0.83 (0.78-0.92) |  |
|  | All iNPH patients | Preoperative (n=20) | 0.89 (0.82-1.02) 100 | 0.85^a^ | 0.81 (0.74-0.92) 97 | 0.73^a^ |
|  |  | Postoperative (n=16) | 0.94 (0.89-1.04) 106 | 0.77^a^ | 0.88 (0.77-0.94) 106 | 0.65^a^ |
|  |  | Change (n=16) | 0.05 (-0.13-0.10) 6 | 0.83^b^ | 0.07 (-0.05-0.13) 9 | 0.57^b^ |
|  | Responders | Preoperative (n=15) | 0.89 (0.85-0.99) 100 | 0.77^a^ | 0.81 (0.75-0.91) 98 | 0.51^a^ |
|  |  | Postoperative (n=12) | 0,94 (0.91-1.07) 107 | 0.52^a^ | 0.88 (0.81-0.94) 106 | 0.43^a^ |
|  |  | Change (n=12) | 0.05 (0.01-0.15) 7 | 0.31^b^ | 0.07 (0.01-0.22) 8 | 0.24^b^ |
|  | Non-responders | Preoperative (n=5) | 0.97 (0,74-1.21) 108 |  | 0.79 (0.73-1.15) 95 |  |
|  |  | Postoperative (n=4) | 0.84 (0.74-1.02) 94 |  | 0.80 (0.67-0.95) 96 |  |
|  |  | Change  (n=4) | -0.13 (-0.22-0.01) -14 |  | 0.01 (-0.26-0.04) 1 |  |

Note: ^b^compared to preoperative values, IQR = Interquartile Range.
